# Serotonin mediates stress-like effects on responses to non-nociceptive stimuli in the medicinal leech *Hirudo verbana*

**DOI:** 10.1242/jeb.243404

**Published:** 2022-06-09

**Authors:** Danielle Mack, Andrew Yevugah, Kenneth Renner, Brian D. Burrell

**Affiliations:** 1 Division of Basic Biomedical Sciences, University of South Dakota, Vermillion, SD 57069, USA; 2 Center for Brain and Behavior Research, University of South Dakota, Vermillion, SD 57069, USA; 3 Department of Biology, University of South Dakota, Vermillion, SD 57069, USA

**Keywords:** Serotonin, Sensitization, Nociception, Endocannabinoid, Stress, *Hirudo verbana*

## Abstract

Noxious stimuli can elicit stress in animals that produce a variety of adaptations including changes in responses to nociceptive and non-nociceptive sensory input. One example is stress-induced analgesia that may be mediated, in part, by the endocannabinoid system. However, endocannabinoids can also have pro-nociceptive effects. In this study, the effects of electroshock, one experimental approach for producing acute stress, were examined on responses to non-nociceptive mechanical stimuli and nociceptive thermal stimuli in the medicinal leech (*Hirudo verbana*). The electroshock stimuli did not alter the leeches’ responses to nociceptive stimuli, but did cause sensitization to non-nociceptive stimuli, characterized by a reduction in response threshold. These experiments were repeated with drugs that either blocked synthesis of the endocannabinoid transmitter 2-arachidonoylglycerol (2-AG) or transient receptor potential vanilloid (TRPV) channel, which is known to act as an endocannabinoid receptor. Surprisingly, neither treatment had any effect on responses following electroshock. However, the electroshock stimuli reliably increased serotonin (5-hydroxytryptamine or 5HT) levels in the *H. verbana* CNS. Injection of 5HT mimicked the effects of the electroshocks, sensitizing responses to non-nociceptive stimuli and having no effect on responses to nociceptive stimuli. Injections of the 5HT receptor antagonist methysergide reduced the sensitization effect to non-nociceptive stimuli after electroshock treatment. These results indicate that electroshocks enhance response to non-nociceptive stimuli but do not alter responses to nociceptive stimuli. Furthermore, while 5HT appears to play a critical role in this shock-induced sensitizing effect, the endocannabinoid system seems to have no effect.

## Abstract (precis)

**Summary:** The role of serotonin and endocannabinoids in mediating the effects of potentially stress-inducing stimuli on *Hirudo verbana*’s response to nociceptive and non-nociceptive input.

## INTRODUCTION

Encounters with noxious stimuli can be stressful and produce both immediate and long-lasting behavioral and physiological adaptations. One type of stress response is increased reactivity to external stimuli with exposure to aversive-to-noxious stimuli producing sensitization of defensive behaviors (Walters et al., 1983; Boulis and Sahley, 1988; Davis, 1974, 1989; Crook et al., 2013; Babcock et al., 2009; Walters et al., 2001). In the context of pain research, stress has been shown to contribute to allodynia, the perception of pain in response to what is normally non-painful stimuli (Bardin et al., 2009; Alexander et al., 2009; Sosanya et al., 2019; Wu et al., 2021). However stress is also well known for reducing the perception of pain, referred to as stress-induced analgesia (SIA) (Butler and Finn, 2009). How can stress produce both pro-nociceptive effects, such as allodynia and anti-nociceptive/analgesic effects? This issue is complicated by the fact that most studies of stress and its effect on pain do not assess both pro- and anti-nociceptive effects at the same time. In this study, we used the annelid *Hirudo verbana* (the medicinal leech) to examine whether pro- and anti-nociceptive effects can be induced by a potentially stress-inducing stimuli. We also examined whether the endocannabinoid system plays a neuromodulatory role in regulating these opposing processes.

The endocannabinoid system consists of lipid neurotransmitters, such as 2-arachidonoylglycerol (2-AG) and anandamide (AEA), both metabotropic (cannabinoid receptors CB1 and CB2) and ionotropic (transient receptor potential vanilloid, TRPV1) receptors, and synthesizing and metabolizing enzymes (Katona and Freund, 2012). There is considerable interest in cannabinoid-based treatments for pain (Maldonado et al., 2016; Hill et al., 2017) and evidence suggests that the endocannabinoid system contributes to stress-induced analgesia (SIA) (Gregg et al., 2012; Atwal et al., 2020; Hohmann et al., 2005). However, the endocannabinoid system can also have pro-nociceptive effects via depression of spinal inhibitory neurons, leading to leading to disinhibition of non-nociceptive afferent input (Pernia-Andrade et al., 2009; Higgins et al., 2013). Disinhibition of non-nociceptor synapses allows these afferents to have access to nociceptive circuitry in the spinal cord and is an important physiological mechanism mediating allodynia (Torsney and MacDermott, 2006; Arcourt and Lechner, 2015; Petitjean et al., 2015). Therefore, it is possible that stress simultaneously mediates SIA through modulation of nociceptive afferents, while also producing sensitizing effects through disinhibition of non-nociceptive afferents.

Studies using *H. verbana* have the potential to address this question. 2-AG and anandamide are present in the *H. verbana* nervous system and there is pharmacological and genetic evidence of synthesizing and metabolizing enzymes (Matias et al., 2001) (Kabeiseman et al., 2020). Although it is not clear if *H. verbana* has an orthologue of the CB1/2 receptors, there is evidence of a TRPV-like channel that serves as a cannabinoid receptor (Yuan and Burrell, 2010). Furthermore, there appears to be a remarkable level of conservation in the endocannabinoid-based modulation between *H. verbana* and vertebrates (Edwards, 2014; Yang et al., 2016; Paulsen and Burrell, 2019). Both 2-AG and AEA depress *H. verbana* nociceptive synapses similarly to spinal C-fiber synapses, but also potentiate non-nociceptive synapses via a disinhibition mechanism, as observed in mammals (Wang and Burrell, 2016; Yuan and Burrell, 2010; Higgins et al., 2013; Pernia-Andrade et al., 2009; Yang et al., 2016; Kato et al., 2012). In addition, both *in vivo* experiments and those using semi-intact preparations have shown that endocannabinoids depress behavioral responses to nociceptive stimulation, but enhance responses to non-nociceptive stimuli (Wang and Burrell, 2018; Hanson and Burrell, 2018; Summers et al., 2017; Yuan and Burrell, 2013). Therefore, studies using *H. verbana* can potentially provide insights as to whether stress-like stimuli can produce both pro- and anti-nociceptive effects and if the endocannabinoid system contributes to these opposing neurobehavioral processes.

## MATERIALS AND METHODS

Medicinal leeches, *Hirudo verbana* Carena 1820, were obtained from a commercial supplier (Niagara Medicinal Leeches, Cheyenne, WY, USA) and weighed approximately 3 g each. The animals were housed in artificial pond water (0.5 g sea salt per liter of water) with incubator temperatures held at 15°C. The incubator cycled through 12 h: 2 h light:dark periods.

Drugs were prepared immediately before injection from frozen stocks added to the *H. verbana* saline solution (in mmol l^−1^: 114 NaCl, 4 KCl, 1.8 CaCl_2_, 1 MgCl_2_, 5 NaOH, and 10 HEPES; pH=7.4). Tetrahydrolipstatin (THL), dimethyl sulfoxide (DMSO), serotonin (5HT), SB366791 and methysergide were obtained from Sigma-Aldrich (St Louis, MO, USA). THL and SB366791 stocks were dissolved in DMSO before freezing. The methysergide and 5HT were dissolved in saline before freezing. Control experiments were performed using injections of saline as well as injections of DMSO. The injections of THL, SB366791, methysergide and control injections of DMSO and saline were carried out immediately before the initial sensory testing and acclimation. Animals were taken out of the incubator and placed in an ice dish with cold pond water for 1 min to reduce movement and sensitivity to stimulation and then injected on their ventral side at the junction of the body segments and the posterior sucker: an approach that has been used effectively in prior experiments (Wang et al., 2015; Summers et al., 2017). To test for the potential role of endocannabinoids, THL, which blocks activity of the 2-AG synthesizing enzyme DAG lipase, was injected into the animals prior to electroshock delivery (100 µl at 50 or 25 μmol l^−1^). Another approach was to inject 25 μmol l^−1^ of SB366791, which blocks the TRPV1 channels. Control injections of DMSO (0.05%) were performed in parallel. To explore the role of 5HT in the experiment, injections of 5HT and methysergide, a 5HT receptor antagonist were applied. In humans, methysergide is nonselective 5HT_1_, 5HT_2_ and 5HT_7_ serotonin receptor antagonist. Injections of 400 μmol l^−1^ methysergide were run in parallel with control injections of saline. Injections of 200 μmol l^−1^ and 500 μmol l^−1^ 5HT were used in place of the electric stimulations. Control injections of saline were run in parallel.

**Fig. 1.**
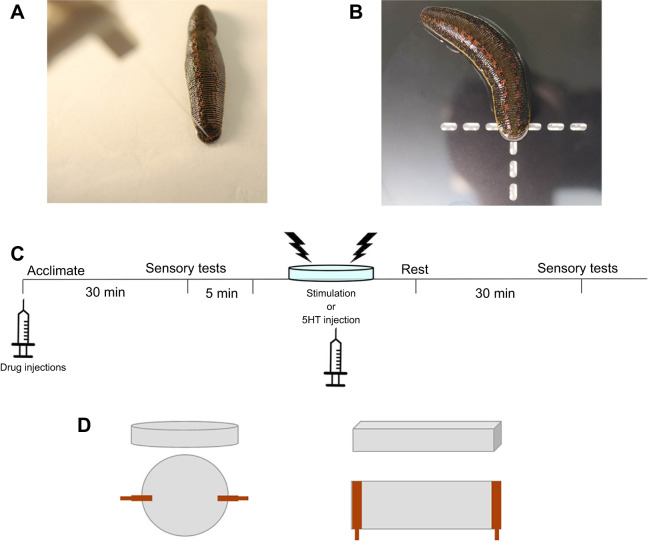
**Behavioral testing methods used for *Hirudo verbana*.** (A) Example of Von Frey fiber stimulation of the *H. verbana* posterior sucker. (B) Example of Hargreaves apparatus targeting the posterior sucker. (C) Experimental timeline for testing the effects of acute stress via electroshock. Note that not all experiments included a drug injection at the start of the experiment. (D) Comparison of the two chamber shapes and the distribution of the leads to deliver shocks. Positive and negative leads are shown in orange. Dimensions are provided in the Materials and Methods.

Changes in response thresholds to non-nociceptive mechanical stimuli was assessed using Von Frey filaments. Von Frey stimuli were applied to the posterior sucker nearest to where the sucker meets the body of the animal (Fig. 1A). The measured response was a localized shortening reflex (defined in the subsequent paragraph). Threshold was assessed using a simplified up-down (SUDO) method (McMackin et al., 2016). In this method, a Von Frey of mid-range strength (in these experiments, 0.07 g) was designated as the starting value. If the animal did not respond to this force, the next largest force was applied after a 60 s waiting period. This pattern continued until the two lowest force Von Frey stimuli to which the leech responded were found. The average of these two values was recorded as the threshold for the animal at that time.

Responses to nociceptive (thermal) stimuli were assessed using the Hargreaves apparatus with the infrared light targeted to the posterior sucker of the animal (Fig. 1B). As with paw-withdrawal behaviors in rodents for which the Hargreaves apparatus was originally designed (Yeomans and Proudfit, 1994), the latency for each leech to withdraw its sucker followed by full body shortening away from the stimulus was measured. A second approach in the delivery of nociceptive stimuli was to use a 25 G needle to poke the posterior sucker. The needle was used to poke the posterior sucker of the leech (in the same region as Von Frey filaments). A total of 10 pokes were administered with each poke occurring every 30 s. Responses to these stimuli were graded based on the behavior elicited; whole body shortening=2, localized shortening reflex=1 and no response=0. In whole-body shortening, all of the body segments contract nearly simultaneously (Kristan et al., 2005). Local shortening, on the other hand, involves the contraction of only a few body segments, the stimulated segment, adjacent segments and perhaps a few more segments that are in close proximity. The 10 responses were summed to a final score. The final score was recorded before and after the electric stimulation protocol. These two values were compared to determine if any behavioral changes occurred.

The timeline for the experiments is depicted in Fig. 1C. Electroshocks were used to produce a stress-like state in *H. verbana*, an approach that has been used in both invertebrates and vertebrates, e.g. (Fossat et al., 2014; Hohmann et al., 2005). Initially, each animal received electroshock stimulations in an 8.5 cm diameter Petri dish filled with 25 ml pond water (Fig. 1D, left). Shocks were delivered using Grass S88 stimulator with a stimulus isolation unit (Astro Med Inc., RI, USA). The voltage used for the experiment was determined by finding the threshold of voltage that elicits a whole body shortening reflex (Shaw and Kristan, 1995) and then increasing that voltage to 50% over that threshold value. Following a 5 min period to adapt to the chamber, the stimulation protocol began. Shocks (1 ms pulse duration) were delivered every 30 s for 15 min, totaling to 30 stimulations. Experiments of only five stimulations were also performed with the same parameters. Owing to concerns that there were regions of the circular chamber where the animal could escape the electroshocks, a box chamber (5×9×2.5 cm) filled with 30 ml pond water replaced the Petri dish in later experiments (Fig. 1D, right), although no differences in the behavioral effects of shocks were observed between the two types of recording chambers (Fig. 1B). Following the electroshock treatment, each leech had a 30 min resting period before retesting response parameters.

### HPLC experiments

To explore potential changes in neurotransmitters within the leech CNS as a result of electroshock treatments, octopamine (OA), dopamine (DA), and serotonin (5-HT) in the *H. verbana* CNS were analyzed using high-performance liquid chromatography (HPLC) with electrochemical detection as described previously with minor modifications (Bubak et al., 2013). Immediately following shock treatment, 20 segmental body ganglia were quickly dissected in an ice-lined dish of filled with ice-cold leech saline. In each animal the ganglia were divided into groups of five and placed into 60 µl of sodium acetate buffer (pH 5.0) containing the internal standard alpha-methyl dopamine (Merck & Co., Inc., Kenilworth, NJ, USA), disrupted by sonication using a 4710 Ultrasonic Homogenizer (Cole-Parmer Instrument Co., Chicago, IL, USA) and stored at −70°C. Prior to analysis, the samples were thawed and centrifuged at 17,000 ***g*** for 15 min. The supernatant was removed and a Waters 17plus autoinjector was used to inject 50 µl of the supernatant onto a C_18_ 4 µm NOVA-PAK radial compression column held at 30°C (Waters Associates, Inc. Milford, MA). The initial mobile phase contained of 8.6 g sodium acetate, 250 mg EDTA, 14 g citric acid, 130 mg octylsulfonic acid and 160 ml methanol in 1 liter of distilled water (all chemicals obtained from Sigma-Aldrich (St Louis, MO, USA) and was subsequently modified by additions of small amounts of acid and 1-octane sulfonic acid to optimize the separation. Electrochemical detection of amines was accomplished using an LC 4 potentiostat and glassy carbon electrode (Bioanalytical Systems, West Lafayette, IN, USA) set at a sensitivity of 5 nA V^−1^ with an applied potential of +0.85 V (+0.995 V when octopamine was included in the analysis) versus an Ag/AgCl reference electrode. The pellet of nervous tissue was solubilized in 100 µl of 0.4 N NaOH and protein content was analyzed using the Bradford method. A Chromatography Station for Windows (CSW32) data program (DataApex Ltd., Czech Republic) was used to determine monoamine concentrations in the internal standard mode using peak heights calculated from standards. Corrections were made for injection versus preparation volumes and sample monoamine concentrations were normalized by dividing pg amine by µg protein to yield pg monoamine µg^−1^ protein.

### Data analysis

Data collected for all experiments were analyzed and graphed using SigmaPlot software (v.12.0). Data from the nociceptive and non-nociceptive responses were compared using the pre-treatment and post-treatment values recorded from the experiment and represented as the percentage change between the values (post-treatment/pre-treatment). Data from the HPLC measurements were grouped by neurotransmitter type. These data were analyzed by grouping the ganglia by 5 as they were dissected or by grouping all the ganglia. Statistical analysis was done using *t*-tests and two-way analysis of variance (ANOVA). *t*-tests were reported using mean and standard error. Student–Newman–Keuls *post hoc* tests were used to determine what groups were different in the ANOVA results. Significance was determined with an α level of *P*≤0.05.

## RESULTS

### Effects of electroshocks on responses to nociceptive and non-nociceptive stimuli

**Fig. 2.**
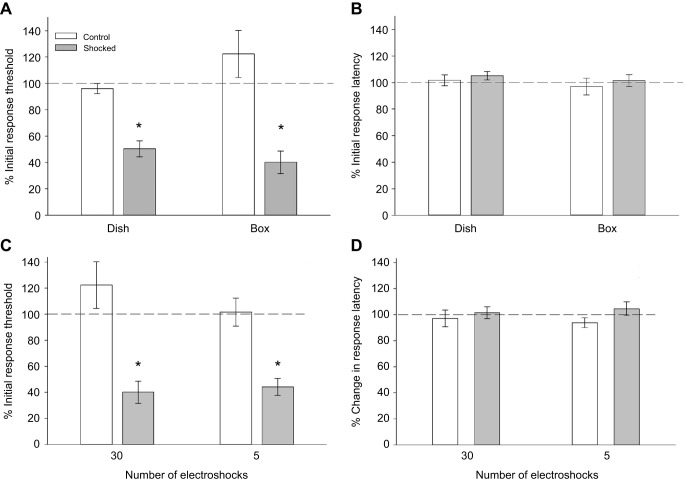
**Comparison of the effects of shock based on delivery in Petri dish versus rectangular box and on number of shocks.** (A) Response thresholds to non-nociceptive mechanical stimulation (Von Frey fibers) are significantly reduced (*P<0.001) in shocked animals (*N*=14) vs. controls that received no shocks (*N*=11). The effects of shocks on responses to non-nociceptive and nociceptive stimuli do not vary in the round (dish; *N*=10) or rectangular (box; *N*=10) chamber. (B) Responses to nociceptive thermal stimuli (Hargreaves apparatus) are not affected by electroshocks regardless of the chamber in which the shocks were delivered. (C) The degree of sensitization to non-nociceptive mechanical stimuli is nearly identical in animals that received 30 shocks (*N*=16) vs. those that received 5 (*N*=16). (D) No change in responses to nociceptive stimuli is observed in either the 30- or 5-shock group.

Electroshocks were used as potential stress-inducing stimuli in *H. verbana*. Sensitization to non-nociceptive mechanical stimuli was observed following delivery of the electroshocks. In the shocked group tested in the round Petri dishes, the response threshold to Von Frey stimuli decreased to 50.3±6.3% (mean±s.e.m.) of the pre-treatment levels (Fig. 2A; *N*=14). In control animals placed in the treatment chambers, but received no shocks (*N*=11), the response threshold was 96±4% of pre-test levels. The average percent change in response threshold to Von Frey stimuli was lower in the shocked group compared with controls (*t*=5.74, *P*<0.001). Shock treatment did not significantly affect responses to nociceptive thermal stimuli delivered by the Hargreaves apparatus (Fig. 2B). The average percentage change in response latency was 105.3±3.2% in the shocked group and 101.7±4.2% in the control group (*t*=−0.68, *P*=0.50).

In round Petri dishes, animals may have avoided electroshock stimulations by retreating to the outer edges where they were not between the leads delivering current to the chamber. To determine if this was an issue, rectangular ‘box’ chambers were developed so that the animals could not avoid the electroshock. The results for the shocked and control groups (*N*=10 for both) shocked in the box chambers were similar to those shocked in the dishes; shocked animals were sensitized to non-nociceptive stimuli, but no change was observed in response to nociceptive stimuli (Fig. 2A,B). A two-way ANOVA examining the non-nociceptive stimulus revealed that there was a significant effect of the shocks in both chambers (*F*_1,44_=41.77, *P*<0.001). There was no significant difference between the type of chamber (*F*_1,44_=0.66, *P*=0.42) and no significant interaction effect between shock and chamber (*F*_1,44_=3.41, *P*=0.07). The results from the Hargreaves apparatus were similar in both the circular and rectangular shock chambers in that electroshocks had no effect on response latency. This was confirmed by a two-way ANOVA that showed no significant effect due to shocks (*F*_1,44_=0.77, *P*=0.39), different chamber shapes (*F*_1,44_=0.87, *P*=0.36) or an interaction effect between shock and chamber shape (*F*_1,44_=0.009, *P*=0.92). These data indicate that the two types of shock chambers produce consistent behavioral effects. Nevertheless, we will indicate whether the box or dish chambers were used in the subsequent experiments.

Because no effect of electroshocks was observed in responses to nociceptive stimuli, there was a concern that the number of electroshocks may have been too many, perhaps fatiguing the animals. Therefore, an alternative electroshock protocol was tested to determine if fewer shocks – 5 shocks over a 2.5 min period (Control *N*=16, Shock *N*=16) – would produce a different behavioral effect. These experiments were performed in the box chamber. As shown in Fig. 2C, the 5-shock and 30-shock protocol produced equivalent levels of sensitization to non-nociceptive stimuli. A two-way ANOVA analysis confirmed that there was a significant difference in the shock group compared with the controls (*F*_1,51_=39.53, *P*<0.001), but that there was no significant difference based on the number of stimulations (*F*_1,51_=0.56, *P*=0.46), nor was there any interaction effect between the number of stimulations and the effect of shocks (*F*_1,51_=1.24, *P*=0.27). As shown in Fig. 2D, the 5-shock and 30-shock protocol were also equivalent in that neither influenced the response latency to thermal nociceptive stimuli. A two-way ANOVA analysis confirmed that there was no significant difference between the shock and control groups (*F*_1,51_=2.18, *P*=0.15), no effect due to the number of stimulations (*F*_1,51_=0.0005, *P*=0.98) and no interaction effect (*F*_1,51_=0.38, *P*=0.54).

**Fig. 3.**
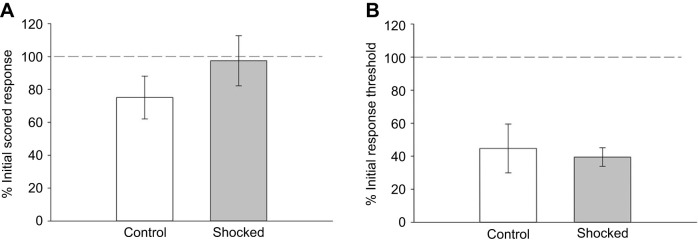
**Effect of electroshocks on responses to mechanical nociceptive stimuli.** (A) No statistically significant difference is observed for scored responses to needle pokes between unshocked, control animals (*N*=7) and shocked animals (*N*=7). (B) Needle pokes did sensitize the control animals to non-nociceptive stimuli in the control group that was equivalent to the effects of the electroshocks. This is an indication that the needle pokes are noxious stimuli.

Since electroshocks did not alter responses to thermal nociceptive stimuli, the possibility that shocks alter responses to mechanical nociceptive stimuli was examined. Like mammals, *H. verbana* possess nociceptors that only respond to mechanical stimuli and polymodal nociceptors that respond to mechanical, chemical and thermal noxious stimuli (Pastor et al., 1996). Response to 10 needle pokes was used as a test of mechanical nociception and took the place of the Hargreaves apparatus tests. Based on published protocols (Hogan et al., 2004; Cowie et al., 2018), pokes from a 25 G needle were used to assess responses to mechanical nociceptive stimuli. Ten needle pokes were applied to the posterior sucker and behavior was scored based on counts of whole-body shortening, local shortening, no response and evasion (see Materials and Methods) prior to and after electroshock stimuli (5-shock procedure using the box chamber). Electroshocks produced no change in the needle poke score (Fig. 3A) as confirmed by a *t*-test which showed no significant difference between the control and shocked groups (*t*=−1.11, *P*=0.29; *N*=7 for both groups). To further establish that the needle pokes represented a noxious stimulus to *H. verbana*, response thresholds to Von Frey fibers were tested following the second series of needle pokes and compared with pre-treatment thresholds. In the shocked group, the response threshold was reduced as expected (39.5±5.7% of initial threshold levels; Fig. 3B). In the control group, which was poked but received no shock, the response thresholds to Von Frey stimulation after the second series of pokes was substantially reduced compared with pre-treatment levels (44.8±14.8%; Fig. 3B). There was no significant difference in the percentage of initial response threshold between the control and shocked groups (*t*=0.33, *P*=0.75). This sensitizing effect of the pokes on the response threshold supports the conclusion that the needle pokes are a noxious stimulus to the animal. Furthermore, the findings that electroshocks do not affect responses to the nociceptive needle pokes is supportive of the results from the experiments using thermal nociceptive stimuli: that electroshock stimuli do not affect responses to subsequent nociceptive stimuli in *H. verbana*.

**Fig. 4.**
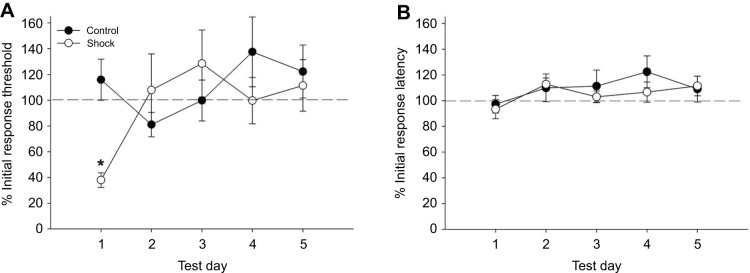
**Lack of long-term effects of electroshocks.** (A) Following a decrease in response threshold to non-nociceptive stimuli on the same day the shocks were delivered (day 1; *P<0.05), threshold returned to initial levels on days 2–5 (*N*=6) compared with control animals (*N*=9). (B) No change in response latency to nociceptive stimuli was observed during the 5-day test period following shocks on day 1.

The duration of the sensitizing effects of the electroshock treatment was assessed over a 5-day period. The 30-shock protocol was used and animals shocked in both the dish and the box chambers were combined based on the results in Fig. 2. Changes in the response threshold to Von Frey fibers and in response latency to thermal nociceptive stimuli were tested on the day of shock treatment (day 1) and once daily for days 2–5. As shown in Fig. 4A, while the expected sensitization to Von Frey stimuli was observed in the shocked group on day 1, the response threshold returned to pre-shock levels on days 2–5. The response threshold in the control group did not change over the testing period. A two-way ANOVA detected no overall effect of treatment (shock) between the control (*N*=9) and the shocked (*N*=6) groups, (*F*_1,84_=1.28, *P*=0.26) and no effect based on the testing day (*F*_4,84_=1.65, *P*=0.17). There was a significant interaction effect between the treatment and testing day (*F*_4,84_=2.59, *P*<0.05) and a Student–Newman–Keuls *post hoc* test confirmed a significant difference between the control and shocked groups on day 1 (*P*<0.05). No changes in responses to the Hargreaves apparatus were observed in either the shocked or control groups over the 5-day testing period (Fig. 4B). A two-way ANOVA showed no effect of treatment (*F*_1,84_=0.62, *P*=0.44), testing day (*F*_4,84_=1.31, *P*=0.28) or interaction effect (*F*_4,84_=0.36, *P*=0.83). From these observations, it is concluded that electroshocks produced no lasting sensitization to non-nociceptive stimuli beyond day 1 and there is no delayed sensitizing effect on responses to thermal nociceptive stimuli.

### Role of endocannabinoid signaling

Endocannabinoid signaling has been shown to play an important role in stress-related behavioral changes (Gorzalka et al., 2008; Lutz et al., 2015). Endocannabinoids in *H. verbana* modulate synaptic transmission by both non-nociceptive and nociceptive afferents (Paulsen and Burrell, 2019). In particular, we have found that high frequency stimulation (HFS) of nociceptive afferents elicits heterosynaptic long-term potentiation (LTP) in non-nociceptive pressure sensory (P) cell synapses that require 2-AG synthesis and activation of a TRPV-like channel (Wang and Burrell, 2018). In addition, endocannabinoids contribute to injury-induced sensitization in *H. verbana* (Jorgensen and Burrell, 2022). We hypothesized that electroshocks stimulate nociceptive afferents, eliciting endocannabinoid-mediated LTP in P synapses, which contributes to sensitization of the Von Frey response threshold. To test this, animals were injected with 100 µl of either 25 µmol l^−1^ THL (control, *N*=9; shock, *N*=10), 50 µmol l^−1^ THL (control, *N*=8; shock, *N*=7), 25 µmol l^−1^ SB366791 (control, *N*=12; shock, *N*=11) or 0.05% DMSO (control, *N*=10; shock, *N*=9). THL blocks activity of diacylglycerol lipase (DAGL), which synthesizes 2-AG and SB366791 blocks TRPV1 channels, which can act as cannabinoid receptors (Cristino et al., 2020; Zygmunt et al., 2013). Injection procedures are based on previous studies (Wang et al., 2015; Summers et al., 2017; Jorgensen and Burrell, 2022).

**Fig. 5.**
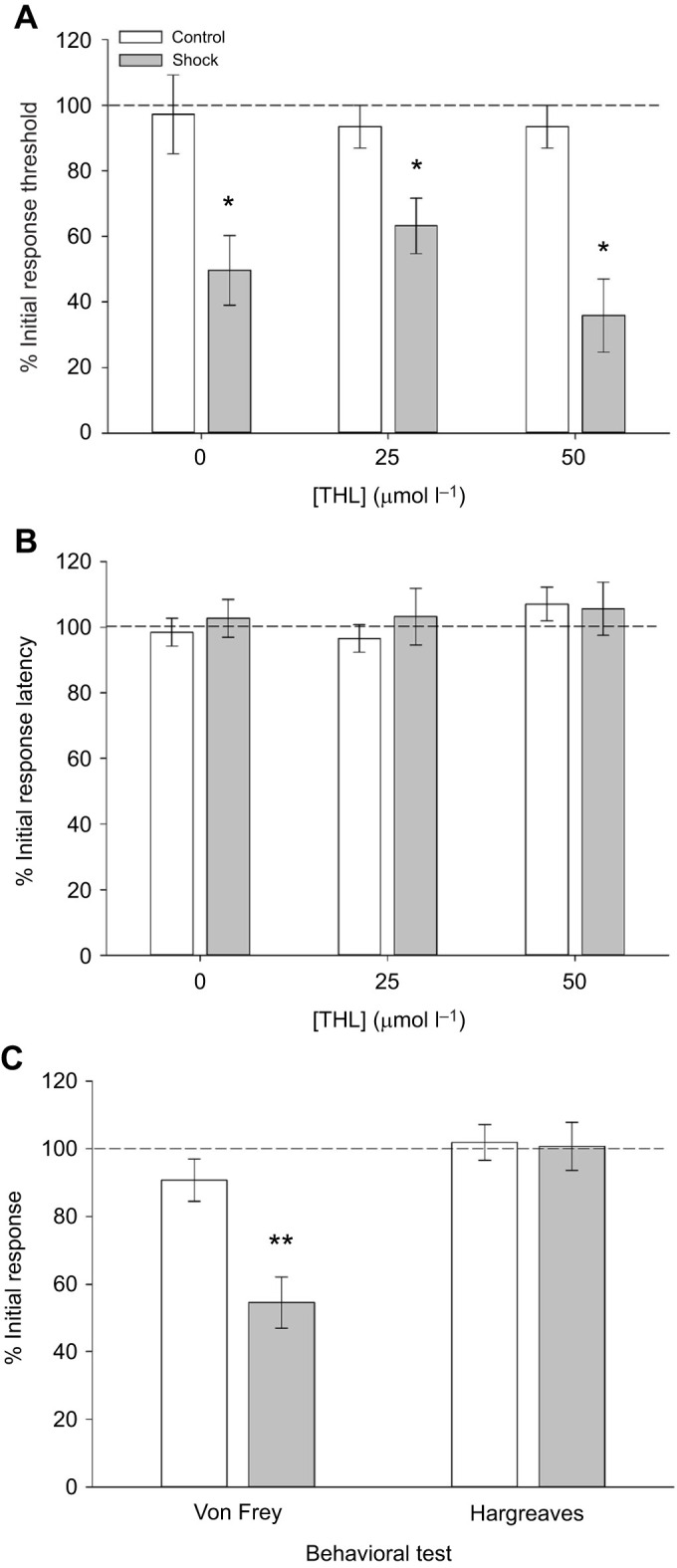
**Inhibition of endocannabinoid signaling does not affect shock-induced sensitization.** (A) Animals injected 25 µmol l^−1^ THL (shocked *N*=10, control *N*=9) or 50 µmol l^−1^ THL (shocked *N*=7, control *N*=8) are still sensitized to non-nociceptive stimuli by electroshocks (*P<0.001) and are no different from DMSO-injected (0; shocked *N*=9, control *N*=10) animals. (B) No shock-induced changes in response to nociceptive stimuli are observed in DMSO- or THL-injected animals. (C) Shock-induced sensitization to non-nociceptive stimuli are still observed in animals injected with SB366791 (shocked *N*=11, control *N*=12). Drug injection has no effect on responses to nociceptive stimuli in shocked animals (**P<0.005). Unshocked animals injected with SB366791 (controls) are not affected by the drug.

As noted in the Materials and Methods, drug injections were carried out 30–35 min prior to the sensory testing and delivery of the electroshocks (Fig. 1C) in ice-cold pond water to chill the animal and minimize any sensitizing effect of the injection itself. These experiments utilized the 30-shock stimulation protocol and were performed in the dish chamber. As shown in Fig. 5A, 25 and 50 µmol l^−1^ THL had no effect on shock-induced sensitization to non-nociceptive stimuli. Response thresholds in the DMSO-injected group and both THL-injected groups were reduced following electroshock treatment, as observed in earlier experiments. DMSO and THL injections did not have any effect on response threshold in the control (non-shocked) groups. A two-way ANOVA detected a significant effect of the shocks (*F*_1,60_=28.35, *P*<0.001). However, there was no significant effect of drug treatment (*F*_2,60_=0.25, *P*=0.78) and no interaction effect between the shocks and drug treatment (*F*_2,60_=0.48, *P*=0.62). As noted earlier, electroshocks did not affect responses to nociceptive thermal stimuli and this lack of effect was also observed in THL-injected animals (Fig. 5B). A two-way ANOVA detected no effect of the shock treatment (*F*_1,60_=0.099, *P*=0.75), drug (*F*_2,60_=0.015, *P*=0.99) or shock×drug interaction (*F*_2,60_=0.12, *P*=0.89).

Injections of the TRPV inhibitor SB366791 were similarly ineffective. Shock-induced sensitization to non-nociceptive stimuli was present in SB366791-injected animals (Fig. 5C). A *t*-test of response thresholds to Von Frey stimulation did detect a significant difference between the control and shocked animals (*t*_21_=3.70, *P*<0.005). No effect of electroshock was observed in responses to nociceptive thermal stimuli in the SB366791-injected animals (Fig. 5C; *t*_21_=0.14, *P*=0.89 between the control and shocked animals). From these results, it is concluded that neither 2-AG nor the *H. verbana* TRPV-like channel mediates the sensitizing effects of electroshock treatment.

One potential concern is that the process of chilling the animals in an ice bath and then injecting them produces additional sensitization that obscures the potential effects of THL and SB366791. One indication of such a sensitizing effect would be a difference between non-injected and DMSO- or drug-injected animals in their initial (baseline) Von Frey response threshold (measured in grams) and response latency to thermal nociceptive stimuli (measured in seconds). These baseline values are shown in Table 1 and one-way ANOVA detected no significant difference in these groups in terms of response threshold (*F*_4,50_=1.13, *P*=0.35) or response latency (*F*_4,50_=0.45, *P*=0.77). These results indicate that the injection procedure did not alter the initial responsiveness of the animals compared with non-injected animals.

**Table 1.**
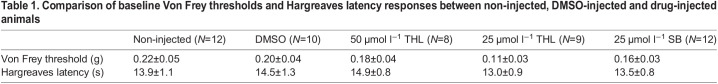
Comparison of baseline Von Frey thresholds and Hargreaves latency responses between non-injected, DMSO-injected and drug-injected animals

### Role of in 5HT during electroshock-mediated sensitization

**Fig. 6.**
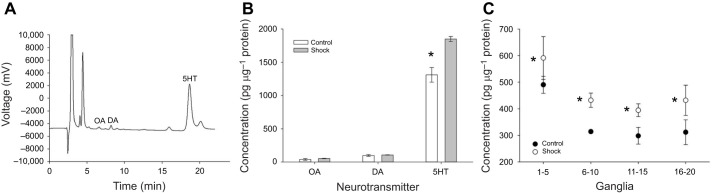
**Shock-induced changes in biogenic amine levels in the *H. verbana* CNS.** (A) Sample chromatogram from *H. verbana* tissue showing a very large peak for 5HT and much smaller peaks for octopamine (OA) and dopamine (DA). (B) Shock treatment (*N*=3) produces a significant increase in 5HT in the CNS compared with controls (*N*=3). (C) Pattern of 5HT levels across the length of the nerve cord. Greatest amounts of 5HT are found in the anterior region (ganglia 1–5), but shock-induced increases in 5HT are observed in all the ganglia sections tested. *P<0.001 indicates a statistically significant effect of shock treatment.

Next, we examined the potential role of biogenic amines in mediating the sensitization effects of electroshock treatment. First, the levels of biogenic amines in the *H. verbana* CNS were measured in control and shock-treated animals. Fig. 6A shows a representative chromatogram of a sample from the *H. verbana* nerve cord. Peaks for octopamine (OA), dopamine (DA) and serotonin (5HT) were detectable, with 5HT being present in particularly large concentrations. Animals underwent the 30-shock protocol in the dish chamber (*N*=3) and biogenic amine levels were compared to nerve cords from control animals (*N*=3). No differences were observed between the control and shocked animals for octopamine (*t*_4_=−0.90, *P*=0.42) or dopamine (*t*_4_=−0.64, *P*=0.56; see Fig. 6B). However, a significant difference was observed in 5HT levels in the CNS between shocked and control animals (*t*_4_=−4.67, *P*<0.01). The 5HT data was further analyzed by region of the CNS, specifically ganglia 1–5, 6–10, 11–15 and 16–20. In both the control and shocked animals, 5HT levels were greater in more anterior regions of the CNS, which is consistent with previous HPLC studies of 5HT content in the *H. verbana* CNS (Lent, 1984). Shock-induced increases in 5HT levels were observed in all sections of the *H. verbana* CNS. A two-way ANOVA detected a significant effect of CNS section (*F*_3,22_=8.60, *P*<0.001) and a significant effect of shock treatment (*F*_1,22_=12.2, *P*<0.005). There was no interaction effect between the segmental location of the ganglia and shock treatment (*F*_3,22_=0.04, *P*=0.99). No differences in dopamine and octopamine levels were observed regarding ganglia location or electroshock treatment (data not shown).

**Fig. 7.**
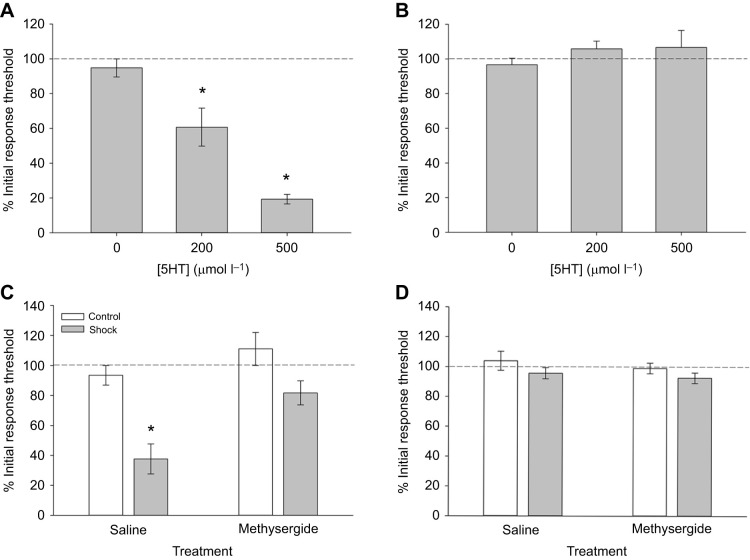
**Role of 5HT in mediating the sensitizing effects of shock treatment.** (A) Injection of increasing concentrations of 5HT produces sensitizing effects on non-nociceptive response thresholds that are similar to the effects of shock treatment (*P<0.005; 0 µmol l^−1^, *N*=10; 25 µmol l^−1^, *N*=10; 50 µmol l^−1^, *N*=11). (B) These same 5HT injections had no effect on responses to nociceptive stimuli. (C) Methysergide reduces the effect of shock treatment (*N*=9) on response threshold to non-nociceptive stimuli compared with saline-injected animals (*N*=9) (*P<0.005). Injection of methysergide (*N*=9) or saline (*N*=13) without shock has no effect on response threshold. (D) Methysergide has no effect on responses to nociceptive stimuli in either the shocked or non-shocked (control) group.

The increases in 5HT levels in the CNS in response to electroshock suggested a potential role for this transmitter during electroshock-induced sensitization to non-nociceptive stimuli. Therefore, *H. verbana* were injected with 200 µmol l^−1^ or 500 µmol l^−1^ 5HT to determine if this neurotransmitter alone could elicit similar changes in behavior. The concentrations of injected 5HT are in the same range as in previous *H. verbana* studies that used ∼3 g animals (Zaccardi et al., 2004; Bisson et al., 2012). Compared with control animals that were injected with saline (*N*=10), both 200 µmol l^−1^ (*N*=10) and 500 µmol l^−1^ (*N*=11) produced a significant decrease in Von Frey response threshold (Fig. 7A; one-way ANOVA, *F*=28.79, *P*<0.001; *post hoc* test, *P*<0.005) and the level of change was similar to that produced by electroshock treatment. A *post hoc* comparison of data from the two groups showed that the response thresholds from the 500 µmol l^−1^ 5HT animals were significantly lower compared with the 200 µmol l^−1^ group. 5HT injections had no effect on the response latencies to the Hargreaves apparatus (Fig. 7B; *F*=0.62, *P*=0.55), again consistent with the effects of electroshock treatment.

The potential role of 5HT in modulating sensitization to non-nociceptive stimuli was further tested by pre-treating leeches with methysergide, an antagonist for 5HT_1_, 5HT_2_ and 5HT_7_ receptors. Four groups of animals were tested following injections of either saline or 400 µmol l^−1^ methysergide: (1) saline (*N*=13), (2) saline+shock (*N*=13), (3) methysergide (*N*=9), and (4) methysergide+shock (*N*=9). Methysergide has previously been used to block behavioral and neurophysiological effects of 5HT in *H. verbana* (Burrell and Sahley, 2005). The electroshock treatment used here was the 5-shock protocol in the box chamber. Although methysergide did not completely prevent shock-induced reduction in response threshold to Von Frey fibers, the sensitizing effect of shocks was substantially reduced (Fig. 7C). A two-way ANOVA of the response threshold data revealed a significant effect of electroshock treatment (*F*_1,43_=19.58, *P*<0.001) and methysergide versus saline injection (*F*_1,43_=10.27, *P*<0.005). There was no significant interaction effect (*F*_1,43_=1.89, *P*=0.18). Methysergide injection had no effect on response latency to thermal nociceptive stimulus (Fig. 7D). A two-way ANOVA found no significant effects of the electroshock treatment (*F*_1,43_=2.94, *P*=0.09), methysergide versus saline injection (*F*_1,43_=0.86, *P*=0.36) or an interaction effect (*F*_1,43_=0.04, *P*=0.84). Collectively, the 5HT and methysergide experiments support the hypothesis that 5HT mediates, at least in part, electroshock-induced sensitization to non-nociceptive stimuli.

## DISCUSSION

Delivery of electroshocks produced a sensitized state in *H. verbana*. Shocked animals were more sensitive to mechanosensory stimuli that elicited the local shortening withdrawal reflex and had increased 5HT content within the CNS. The shock-induced behavioral effects appeared to be mediated, at least in part, by 5HT. Serotonin injections mimicked the effects of electroshocks by inducing a sensitization to non-nociceptive stimuli with no change in response to nociceptive stimuli. Furthermore, electroshock-induced sensitization to non-nociceptive stimuli could be inhibited by the 5HT receptor antagonist methysergide.

Our results using *H. verbana* as a model are consistent with studies of other vertebrate and invertebrate animal models in which 5HT plays a role in stress-related behaviors (Fossat et al., 2015, 2014; Curran and Chalasani, 2012; Adjimann et al., 2021; Amat et al., 1998; Pereira et al., 2019; Bubak et al., 2014, 2020). There is also considerable evidence of 5HT mediating sensitization to somatosensory stimuli in a variety of invertebrate species, including *Aplysia* sp., *H. verbana*, *Tritonia* sp. and *Helix* sp. (Abramova et al., 2006; Barbas et al., 2003; Ehrlich et al., 1992; Katz, 1998). In *H. verbana*, 5HT has been shown to contribute to sensitization of swimming behavior, the local bending reflex, and the whole-body shortening reflex (Ehrlich et al., 1992; Zaccardi et al., 2004; Lockery and Kristan, 1991). In the case of whole-body shortening, 5HT increases the excitability of an interneuron critical for sensitization (the S interneuron) (Burrell et al., 2001; Burrell and Crisp, 2008; Burrell and Sahley, 2005; Modney et al., 1997). It should be noted that local shortening and whole-body shortening in *H. verbana* utilize distinct neural circuits, albeit with some overlap of sensory and motor elements (Wittenberg and Kristan, 1992a,b; Shaw and Kristan, 1995). Sensitization of local shortening has not been studied previously; therefore, distinct 5HT-mediated modulatory processes may be involved.

Although the role of 5HT in this shock-mediated sensitization to non-nociceptive stimuli was not unexpected, we were surprised that endocannabinoid modulation appeared to play no role. Neither THL, which inhibits 2-AG synthesis, nor SB366791, which inhibits the TRPV1 channels had any effect on stress-induced sensitization. These results were unexpected since our previous *in vivo* work showed that injections of 2-AG reduced the threshold to non-nociceptive Von Frey stimulation (Summers et al., 2017) and mediated injury-induced sensitization to these same stimuli (Jorgensen and Burrell, 2022). We have also observed 2-AG/TRPV-mediated behavioral sensitization and synaptic potentiation of non-nociceptive pressure (P) sensory neurons following nociceptor activation in semi-intact preparations and isolated ganglia (Wang and Burrell, 2018; Wang and Burrell, 2016; Higgins et al., 2013). We have hypothesized that there are parallel endocannabinoid- and 5HT-mediated mechanisms for sensitization (Wang and Burrell, 2018). 5HT-mediated sensitization in *H. verbana* may be mediated by changes in interneuron excitability (Burrell and Sahley, 2005), while endocannabinoids appear to act by disinhibition of P cell synapses (Higgins et al., 2013; Wang and Burrell, 2016; Paulsen and Burrell, 2022). It does not appear that the ice-bath and drug injection protocols produced additional sensitization that somehow interfered with the potential effectiveness of THL or SB366791. A previous study using the same drugs and injection methods also did not produce any significant sensitizing effects in *H. verbana* (Hanson and Burrell, 2018) and the 5HT and methysergide experiments in the current study utilized the same injection protocol.

Our interpretation of these findings is that different types of noxious stimuli can elicit different forms of non-nociceptive sensitization mediated by distinct cellular mechanisms. In the present study, electroshock-induced sensitization involved serotonergic neuromodulation and was cannabinoid independent. However, a recent study from our group has found that injury can also induce non-nociceptive sensitization that differs from the shock-induced induced behavioral effects observed here (Jorgensen and Burrell, 2022). Specifically, it is longer lasting, persisting for at least 4 days, and is mediated by 2-AG/TRPV signaling. Exactly how these two different forms of noxious stimuli engage such physiologically distinct forms of sensitization is unknown at this time. It is somewhat surprising that the repeated shock treatment used here did not produce a more persistent form of sensitization, given the extensive work in *Aplysia* sp. demonstrating long-term sensitization following repeated noxious stimuli (often shocks) that was 5HT mediated (Pinsker et al., 1973; Castellucci et al., 1986; Bailey and Chen, 1988). However, it is well established that massed delivery of training stimuli is less effective in forming long-term changes in behavior compared with training that has spaced intervals (Kandel et al., 2014; Yin and Tully, 1996). It is likely that the delivery of the shocks in the current study represents a form of mass training.

Electroshocks did not produce a decrease in the response to nociceptive stimuli analogous to SIA. Neither thermal nor mechanical nociceptive stimuli induced by needle pokes were affected during these stress-like conditions. There are several potential explanations for these observations. One possibility is that *H. verbana* are not capable of stress-induced anti-nociception. However, studies in other invertebrates have demonstrated a reduction in nocifensive behaviors following stress. In the snail, *Cepaea nemoralis*, both heat and cold stressors induced anti-nociceptive effects with the heat stressor effect being blocked by opioid antagonists while the cold stressor effect was opioid independent (Kavaliers, 1987). Opioid-based modulation was also implicated in stress-mediated (tail pinch) anti-nociception in the slug *Arion ater* (Dalton and Widdowson, 1989). Another possible explanation is that the noxious stressor (electroshocks) used throughout the experiments was not well suited for producing an anti-nociceptive effect. Attempts were made to change the number of shocks that were delivered and the geometry of the chamber where the shocks were delivered, but none of these manipulations changed the pattern of behavioral observations. It is possible that the electrical stimulation was too intense for the animal or that the shocks needed to be delivered over a longer period. Introducing multiple trials and/or multiple test days of varying length and intensity into the experimental protocol may produce different behavioral results. Another possibility is that the thermal stimulus delivered by the Hargreaves apparatus may not be an effective approach in measuring analgesic-like effects in *H. verbana*. This seems unlikely since the Hargreaves apparatus has been used to measure anti-nociceptive effects produced by habituation (Hanson and Burrell, 2018) and nociceptive sensitization due to prior injury (Jorgensen and Burrell, 2022). Furthermore, experiments using needle pokes as a mechanical nociceptive stimulus were also unchanged by the electroshock treatment. Finally, it is possible that the series of electroshock stimuli used in this study was not an appropriate stressor. Electroshocks as a stressor were chosen based on previous studies in crayfish and rats (Hohmann et al., 2005; Fossat et al., 2014). The fact that shock-induced sensitization involved 5HT-dependent modulation is consistent with other studies of stress, but is not necessarily a defining feature of stress. One direction for future experiments would be to utilize a more ethologically appropriate stressor, such as exposure to a potential predator or being temporarily placed in a dry environment.

The finding that we could elicit a form of non-nociceptive sensitization that is 5HT dependent, but does not involve endocannabinoids is interesting. As noted earlier in the Discussion, we have a number of observations in which noxious stimuli or direct nociceptor activation elicits sensitization that is endocannabinoid dependent. This includes the observation that injury produces non-nociceptive sensitization that is mediated by endocannabinoids in *H. verbana* (Jorgensen and Burrell, 2022). Future studies should focus on how different noxious stimuli can activate distinct modulatory processes – mediated by 5HT versus endocannabinoids – to produced adaptive sensitization to non-nociceptive stimuli.
